# Simulation Study of Low-Dose 4D-STEM Phase Contrast Techniques at the Nanoscale in SEM

**DOI:** 10.3390/nano15010070

**Published:** 2025-01-04

**Authors:** Zvonimír Jílek, Tomáš Radlička, Vladislav Krzyžánek

**Affiliations:** Institute of Scientific Instruments of the Czech Academy of Sciences, Kralovopolska 147, 61200 Brno, Czech Republic; jilek@isibrno.cz (Z.J.); krzyzanek@isibrno.cz (V.K.)

**Keywords:** electron microscopy, phase contrast, STEM, SEM, 4D-STEM, ptychography, ePIE, iCOM, low dose, carbon nanotubes

## Abstract

Phase contrast imaging is well-suited for studying weakly scattering samples. Its strength lies in its ability to measure how the phase of the electron beam is affected by the sample, even when other imaging techniques yield low contrast. In this study, we explore via simulations two phase contrast techniques: integrated center of mass (iCOM) and ptychography, specifically using the extended ptychographical iterative engine (ePIE). We simulate the four-dimensional scanning transmission electron microscopy (4D-STEM) datasets for specific parameters corresponding to a scanning electron microscope (SEM) with an immersive objective and a given pixelated detector. The performance of these phase contrast techniques is analyzed using a contrast transfer function. Simulated datasets from a sample consisting of graphene sheets and carbon nanotubes are used for iCOM and ePIE reconstructions for two aperture sizes and two electron doses. We highlight the influence of aperture size, showing that for a smaller aperture, the radiation dose is spent mostly on larger sample features, which may aid in imaging sensitive samples while minimizing radiation damage.

## 1. Introduction

The scanning electron microscope (SEM) is widely recognized for its capability to observe the surface morphology of samples using backscattered electron (BSE) detectors and secondary electron (SE) detectors with a focused electron beam [1,2]. However, this technology extends beyond mere visualization; it also enables the application of analytical methods to determine the chemical composition of materials [3,4], using techniques e.g., energy-dispersive X-ray spectroscopy (EDX) or Auger electron spectroscopy (AES).

While SEM provides high spatial resolution for analyzing complex nanostructures, it lacks the surface sensitivity characteristic of X-ray photoelectron spectroscopy (XPS). XPS excels in examining the outermost layers of materials, making it indispensable for detailed surface chemistry analysis [5]. Additionally, the combination of optical tweezers and atomic force microscopy (AFM) offers unique capabilities, allowing for real-time manipulation and observation of individual objects. This setup provides invaluable insights into their interactions and dynamics at a microscopic level, as well as their nanomechanical properties [6]. In contrast, the superior spatial resolution and relatively high-speed imaging of SEM, particularly when used in transmission mode, enables a more comprehensive understanding of both surface and internal morphology and composition of materials.

SEM is often employed in transmission mode for samples that exhibit weak interactions with the electron beam, utilizing images obtained in bright field (BF) and dark field (DF).

Imaging weakly interacting samples using standard methods can be challenging [7]. When an electron beam passes through such samples, the intensity is minimally affected, but the phase of the electron wave changes. Consequently, most of the information from the sample is encoded in these phase changes, making techniques for phase contrast imaging highly desirable [8].

The image obtained using a small BF detector in scanning transmission electron microscope (STEM) mode is almost identical to the phase contrast in a transmission electron microscope (TEM) [9]. However, because the BF detector processes only a selected part of the diffraction pattern by integrating it, it cannot extract all the information in the diffraction pattern, which is essential for optimising dose efficiency. A promising alternative is the use of 4D-STEM techniques. 4D-STEM techniques involve capturing and analyzing the entire diffraction pattern at each position of the electron beam on the sample (these techniques analyze four-dimensional datasets, hence the name). This approach enables the acquisition of detailed information about the sample’s structure. Phase contrast 4D-STEM methods primarily include iCOM (integrated center of mass) [10] and ptychography [11,12]. With advancements in 2D pixel detector technology, along with improvements in their parameters and cost reductions, these methods are becoming accessible for applications in SEM as well.

Samples that weakly interact with the electron beam are often sensitive to the passing energetic electrons and can withstand only a certain amount of radiation dose. For sensitive materials, it is crucial to minimize this radiation dose [13]. In this regard, direct electron detectors [14] provide significant assistance by allowing for the rapid acquisition of entire diffraction images. This reduces the time required for data collection, thereby minimizing the deposition of impurities that could diffuse onto the sample. An additional advantage of these detectors is that they record diffraction patterns with minimal noise, enabling very low electron doses in individual diffraction images. This facilitates the application of fractionalization properties in ptychography [15].

Weakly scattering samples tend to produce low signal and low contrast images, contrast can be aided by staining the sample with contrast agents [16,17]. However, staining has the disadvantage of imaging only a replica and may also damage the sample [18], as contrast agents are typically heavy metals. Stain-free imaging is less invasive but must manage lower signal. Phase contrast is well suited for imaging weakly scattering stain-free samples as it provides sufficient contrast and is dose efficient [19].

The main goal of this article is to demonstrate, through microscopic simulations, what results can be expected from integrating 4D-STEM techniques into a standard high-end SEM with an immersion objective that achieves an optimal resolution of approximately 0.5nm in BF-STEM and 0.3nm in DF-STEM mode, according to [20].

## 2. Materials and Methods

### 2.1. 4D-STEM in SEM Integration

Integrating 4D-STEM techniques into a SEM and high-resolution STEM (HR-STEM) is not identical. In the case of HR-STEM, a projection system allows for the imaging of the back focal plane on the detector and the correction of beam rotation due to the magnetic field. Conversely, these tools are absent in SEM, resulting in the diffraction image being generated exclusively by far-field diffraction. However, this method is limited by the camera distance, which is determined by the geometrical distance between the sample and the detector, approximately 240mm in our case.

**Figure 1:**
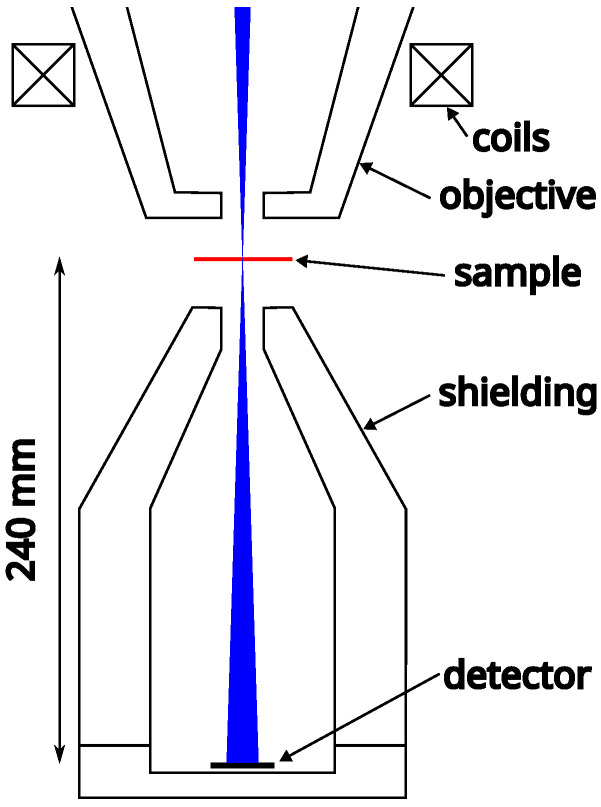
Schematic representation of an electron beam passing through the sample in SEM with magnetic shielding below the sample that allows the beam to pass freely without significant influence from the magnetic field beneath the sample.

The SEM achieves ultimate resolution, especially when utilizing an immersion magnetic objective. However, in this case, the magnetic field is present not only above the sample but also penetrates behind it. While the field behind the sample is insignificant for standard analyses using SE or BSE (note that an immersion field above the sample will affect the SE and BSE trajectories), it can alter the formation of diffraction patterns on the detector, posing a major obstacle for 4D-STEM techniques. Therefore, it is necessary to either use modes without a magnetic field on the sample or to shield the immersion field between the sample and the detector using a magnetic material pole as illustrated in Figure 1. Since such an element also affects the magnetic field before the sample and thus the properties of the primary beam, it is essential to position the pole accurately relative to the SEM objective. This article, however, will not address the specific implementation of the pole or its effects on the primary beam. This problem is represented only by a spherical aberration coefficient (Table 1) and the assumption that the magnetic field does not influence the electrons beneath the sample.

To integrate 4D-STEM techniques into a SEM, it is essential to use a sample stage that does not occupy most of the space beneath the sample. One option is to use a specialized sample stage from microscope manufacturers (such as the CompuStage from Thermo Fisher Scientific), or to replace the standard sample stage with a custom-designed solution that better meets the specific requirements of the experiment.

A crucial part of the 4D-STEM microscope is the pixelated detector. Due to the recent development of pixelated detectors, statistical noise appears to be the dominant factor [21]. This means that direct electron detectors are capable of detecting individual electrons. A Timepix3 [22] is an example of such a detector, capable of capturing an image in 0.8ms, when operated in frame-based mode. It can also be operated in an event driven mode, which accommodates $40\;\mathrm{Mhits}\cdot s^{-1}{\mathrm{cm}}^{-2}$. Timepix3 was considered in this study. The number of pixels of this detector and its pixel pitch are shown in Table 1.

### 2.2. Simulations

To investigate the 4D-STEM imaging capabilities of the microscope described in the last section, we performed microscopic simulations using the software abTEM [23] to obtain simulated 4D-STEM datasets.

In abTEM, we define an atomic model of the sample and then we propagate an electron probe through it. The electron wave is then propagated to the detector, where it is detected. The propagation step through the sample is the key part and is implemented using a multislice algorithm [20]. A simplified (assuming a thin sample) description of propagation of the probe through the sample and its detection follows.

We generate an aberration surface to form the probe by defining the phase error, χ(k), from the ideal unaberrated wave in the reciprocal space. This is done by equating aberration polynomial. In the case of defocus $-C_{10}$ and spherical aberration of third order $C_{30}$, the phase error is given by:

(1) $$\chi \left(\vec{k}\right)=\frac{2\pi }{\lambda }\left(\frac{1}{2}C_{10}\lambda^{2}k^{2}+\frac{1}{4}C_{30}\lambda^{4}k^{4}\right),$$

where λ is wavelength of the electron and $\vec{k}=\left(k_{x},k_{y}\right)$ is a spatial frequency. The probe $\psi_{\mathrm{in}}$ is then calculated as

(2) $$\psi_{\mathrm{in}}\left(\vec{r}\right)=F\left\{A\left(\vec{k}\right)e^{-i\chi (\vec{k})}\right\}\left(\vec{r}\right),$$

where the aperture function $A(\vec{k})$ defines the shape of the aperture. For a circular aperture, $A(\vec{k})$ is determined by its opening semiangle, $\alpha_{a}$. F denotes a Fourier transform.

The propagation of the probe through the sample can, in the simplest case, be modeled as its multiplication by the transmission function $t(\vec{r})$. Consequently, the wave below the sample is given by

(3) $$\psi_{\mathrm{out}}\left(\vec{r}\right)=\psi_{\mathrm{in}}\left(\vec{r}\right)t\left(\vec{r}\right).$$

The transmission function can be defined like

(4) $$t\left(\vec{r}\right)=e^{i\varphi (\vec{r})},$$

where the phase change $\varphi (\vec{r})$ can be related to the projected potential $\Phi_{z}\left(\vec{r}\right)$ by $\varphi \left(\vec{r}\right)=\sigma \Phi_{z}\left(\vec{r}\right)$, where σ is interaction parameter $\sigma =2\pi me\lambda /h^{2}$. In this expression, *m*, *e* and λ is mass, charge and wavelength of the electron, respectively, while *h* is Planck’s constant. Given electric potential $\Phi (\vec{r})$ projected potential is calculated as $\Phi_{z}\left(\vec{r}\right)=\int \Phi \left(\vec{r}\right)dz$. Propagation of the exited wave $\psi_{\mathrm{out}}$ to the detector plane is modeled as

(5) $$\psi_{\det}\left(\vec{k}\right)=F\left\{\psi_{\mathrm{out}}\left(\vec{r}\right)\right\}\left(\vec{k}\right).$$

The intensity ${\left|\psi_{\det}\right|}^{2}$ of the wave at the detector $\psi_{\det}$ is then detected by the given detector. To simulate a finite electron dose in the diffraction patterns, Poisson noise can be applied to the intensity on the detector.

### 2.3. iCOM

Integrated center of mass is a phase contrast technique that requires a focused beam. When a focused beam scans a sample, the mean direction of the beam slightly changes according to the local properties of the sample. This change in direction is called the center of mass (COM), which can be used to extract projected electric field $\vec{E_{z}}\left(\vec{r}\right)$ in the sample. Solving for the projected potential from electric field involves integration, hence the name of the iCOM.

As shown in [24], the iCOM measurement relates linearly to the object phase $\varphi (\vec{r})$:

(6) $$F\left\{I^{\mathrm{iCOM}}\left(\vec{r}\right)\right\}\left(\vec{k}\right)=\frac{1}{2\pi }\bar{F\{{\left|\psi_{\mathrm{in}}\left(\vec{r}\right)\right|}^{2}\}}\left(\vec{k}\right)F\left\{\varphi \left(\vec{r}\right)\right\}\left(\vec{k}\right).$$

A thickness of the sample for which this relation was derived is limited by the validity of the multiplication approximation (3). COM measurement is ideally obtained using a pixelated detector. Nevertheless, one can estimate COM by using a four-quadrant detector, and the resulting technique is called differential phase contrast (DPC) [25,26].

### 2.4. Ptychography

Ptychography is a computational phase contrast imaging technique. A specific ptychographic algorithm processes 4D-STEM data and recovers the image. The first historically developed ptychographic algorithms were direct [27]. These were difficult to apply due to the need to process large amounts of data, which posed a technical challenge at the time. The large amount of data resulted from the need for fine sampling in both direct and reciprocal space. Later, iterative algorithms were developed that reduced the amount of data required for performing ptychographic reconstruction. In particular, the requirements for sampling in direct space were relaxed at the cost of increased complexity of the iterative algorithms. Iterative reconstructions improved significantly after developing algorithms that also reconstructed the probe. Later development in ptychography made it possible to obtain 3D sectioning of the sample with multi-slice ptychography [28] and to mitigate the effect of partial coherence using multi-modal ptychography [29].

In this paper, we will use an iterative algorithm ePIE [30], which can be viewed as an optimization algorithm that starts with an estimate of the probe, propagates the probe through the first guess of the sample, and down to the detector, similarly to how we have described propagation of the probe in Section 2.2. The estimated wave at the detector is then modified by replacing the intensity of the wave with measured intensity at the current scan position while leaving the wave’s phase from the estimate. The resulting wave is propagated back to the sample, where with the new updated exit wave we can update our estimate of both the object and the probe. Iterating this process over all scan positions several times reconstructs the object and the probe from the 4D-STEM data.

In ptychography, the resolution of the reconstruction is not limited by the probe size or the scan step size, but rather by the maximal scattering angle it can successfully process [31]. We can deliberately defocus the probe, which offers several advantages. One of these is that the total intensity of the beam is not concentrated in a sub-nanomer probe, which can reduce radiation damage [15]. The ability to spread the radiation over several scanning positions while still being able to add the contributions from different exposures is called dose fractionation. Another advantage of using a defocused probe is that with a larger probe, we have more significant overlap between the probes as we scan the sample, which is important for obtaining a robust reconstruction [32].

There is a limit to the size of the probe, as for a very large probe the detector will not be able to properly sample all the information in the diffraction pattern. To set this correctly, one should avoid making the size of the probe larger than half of the calculation field of view $\frac{\lambda }{\Delta \alpha }$, where Δα is the angular pixel size of the detector. Nevertheless, ptychography can handle even the case where the probe is too large [33], but this complication can be easily avoided when taking the data.

### 2.5. Coherence Consideration

Both iCOM and ptychography require a coherent beam, making spatial and temporal incoherence a complication to these techniques. A reduction of coherence leads to blurring of features in diffraction patterns and can decrease contrast or resolution.

Spatial incoherence results in broadening of the probe due the effective source size, which can be moderated with small enough currents. Temporal incoherence causes probe broadening due to chromatic aberration. This broadening can be addressed with a cold-field emission gun, a chromatic aberration corrector or an electron monochromator.

In this paper incoherence effects aren’t included, we are assuming ideally coherent electron beam, but they can present a problem if not addressed especially at atomic resolution [34,35]. We also assume that the microscope is stable and that there is no drift of the sample.

It is interesting to note, that ptychography has high resilience to temporal incoherence. The retention of information about the sample’s phase in diffraction patterns is fairly good even with the presence of temporal incoherence [36].

### 2.6. Evaluation: CTF and FRC

Phase contrast imaging techniques can be characterized by a contrast transfer function (CTF), which is defined as

(7) $$\mathrm{CTF}\left(\vec{k}\right)=\frac{F\left\{I\left(\vec{r}\right)\right\}\left(\vec{k}\right)}{F\left\{\varphi \left(\vec{r}\right)\right\}\left(\vec{k}\right)},$$

where $I(\vec{r})$ is the image produced by a given technique and $\varphi (\vec{r})$ is the phase of a sample. For linear imaging techniques, CTF is sample-independent, and thus the technique is well characterized by this quantity.

The CTF of iCOM can be analytically calculated for a given probe, $\psi_{\mathrm{in}}\left(\vec{r}\right)$:

(8) $${\mathrm{CTF}}_{\mathrm{iCOM}}\left(\vec{k}\right)=\frac{1}{2\pi }\bar{F\{{\left|\psi_{\mathrm{in}}\left(\vec{r}\right)\right|}^{2}\}}\left(\vec{k}\right).$$

From this CTF, we can immediately see that the term $F\left\{{\left|\psi_{\mathrm{in}}\left(\vec{r}\right)\right|}^{2}\right\}\left(\vec{k}\right)$ transforms into a convolution of $A\left(\vec{k}\right)e^{-i\chi (\vec{k})}$ and its complex conjugate. Thus, in the case of circular aperture CTF will be nonzero only below the spatial frequencies which correspond to $2\alpha_{a}$.

For ptychography, the CTF is algorithm-dependent [37]. For example direct ptychographical algorithm SSB has a CTF that is strictly limited below spatial frequency corresponding to $2\alpha_{a}$ as it processes only the data inside the bright field disc [38]. And it has been demonstrated [8] that ePIE algorithm can recover the phase of the sample with nearly ideal

(9) $$\mathrm{CTF}(\vec{k})=1.$$

Ideal CTF of ePIE also means, that this algorithm can process diffraction data beyond the bright field disc and recover details which contributed to these higher scattering angles, giving rise to super-resolution [11]. To achieve this it is nevertheless necessary to have a good signal-to-noise ratio in the diffraction pattern and to have a sufficiently coherent beam. Super-resolution capability is sample dependent. A weakly scattering sample may produce very low signal outside the BF disc, whereas a strongly scattering sample, under a similar dose, might scatter enough outside the BF disc to enable super-resolution.

For the evaluation of quality of the reconstruction, a Fourier ring correlation (FRC) [39] can be used. FRC measures the degree of correlation between two images as a function of spatial frequency. In our case, we will compare the ground truth image from simulations to reconstructed image. FRC can be used to estimate the resolution of the reconstructed image based on, for example, FRC=0.143 criterion [40].

## 3. Results

### 3.1. ePIE CTF on an Amorphous Material

To examine how the CTF behaves for ePIE under finite dose we performed simulations of 4D-STEM dataset on an amorphous material. The model of the amorphous material was generated using a transmission function $t(\vec{r})$ with a magnitude set to unity and a phase $\varphi (\vec{r})$, which Fourier components have a constant amplitude and phase set randomly with a uniform distribution. The amplitude was chosen so that only negligible amount of electrons will be scattered outside of the bright field disk, as this emulates weakly scattering sample. The fact that the amplitude is nonzero for every spatial frequency helps with the calculation of CTF, as it prevents divergence.

The CTF will be calculated using (7), as we know the true value of the phase $\varphi (\vec{r})$ from the simulation, and we will obtain the phase image from the ePIE reconstruction. In the ePIE reconstructions, the initial probe was assumed to be known and was kept constant during the reconstruction.

Simulation parameters are listed in Table 2, where, for each aperture semiangle $\alpha_{a}$, the defocus was set to expand the probe to about 40% of calculation field of view. Scan step size was set to $0.1\;d_{95}$, where $d_{95}$ is the diameter of the probe determined by the criterion that 95% of the current lies within the circle of $d_{95}$ diameter. The electron dose was set sufficiently high to ensure successful reconstruction, as the purpose of simulating this dataset was solely to obtain the CTF.

**Figure 2:**
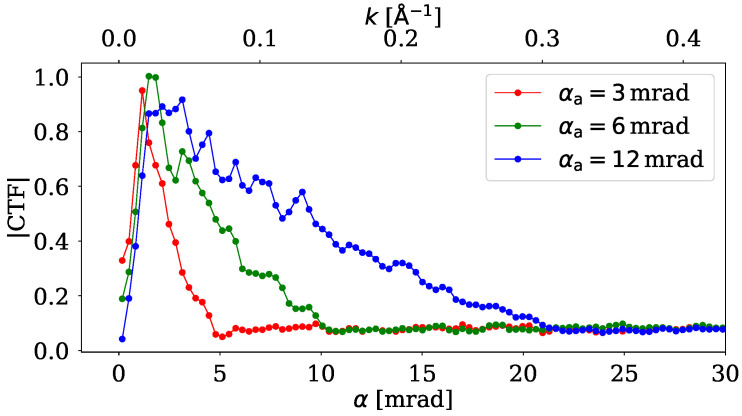
Azimuthal average of |CTF| of ePIE from reconstructions of amorphous sample at finite dose. The CTF was obtained using (7).

Figure 2 shows the azimuthal average of $\left|\mathrm{CTF}\right|$ of the ePIE reconstruction of an amorphous sample for three different aperture semiangles. From these curves, we can see that the CTF quickly increases at low spatial frequencies and then falls to zero as we approach spatial frequencies corresponding to $2\alpha_{a}$. This is because ePIE could process only the data inside a bright field, as the sample was constructed to scatter very weakly outside of it. As in [8], we can conclude that by adjusting the aperture angle, we can tune the technique to be sensitive to the details of our interest.

### 3.2. iCOM CTF on an Amorphous Material

To investigate the CTF of iCOM we repeated the simulation of 4D-STEM data on the amorphous sample but with a focused probe and a very fine scan step size. The step size was set so that spatial frequency corresponding to scattering at the edge of the detector is properly sampled according to Nyquist sampling theorem. The defocus was set for a given aperture angle to minimize phase error χ. To obtain the CTF of the iCOM technique, we simulate a 4D-STEM dataset without statistical noise, corresponding to an infinite dose. By doing so, we can observe how the CTF of iCOM is limited, independent of the effects of a finite dose. All parameters for each aperture angle are in Table 3. The iCOM reconstructions were calculated using the software py4DSTEM [41].

**Figure 3:**
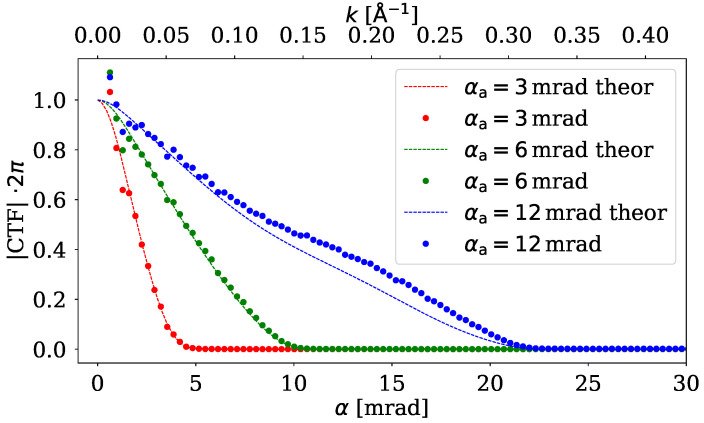
Azimuthal average of |CTF|2π of iCOM calculated for different aperture angles. Dots represent data calculated using (7) from the amorphous sample and lines are calculated using the theoretical equation (8). The regression coefficient ($R^{2}$) of theoretical curve and data points is 0.978, 0.987 and 0.962 for aperture semiangles 3mrad, 6mrad and 12mrad, respectively.

Figure 3 shows the obtained azimuthal average of $\left|\mathrm{CTF}\right|$ multiplied by 2π. The multiplication factor is included because, according to the theory (8), after this multiplication, the result depends only on the probe. Together with the computed CTF from the simulation, the theoretical curve is also included in the figure. These curves are fairly close to each other and we can see that they start to approach zero as we near the spatial frequency which corresponds to $2\alpha_{a}$. This behavior is quite similar to the one we observed for ePIE.

### 3.3. Phase Contrast on a Sample with Carbon Nanotubes

**Figure 4:**
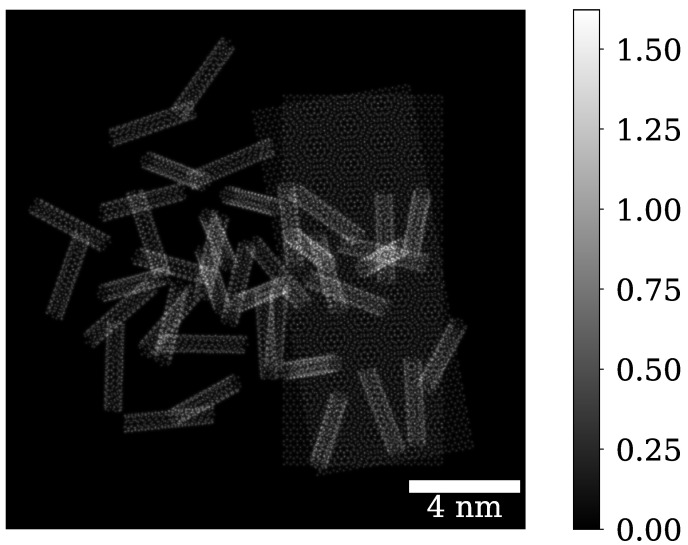
The phase of the sample, consisting of two overlapping graphene sheets and a group of overlapping nanotubes, calculated as a projected potential multiplied by σ.

In this part, we will demonstrate how ePIE and iCOM perform on a sample consisting of several overlapping nanotubes and two overlapping graphene sheets on the right. The corresponding phase of the sample φ (projected potential multiplied by σ) is shown in Figure 4. Here we present results for an aperture semiangle of 3mrad and 12mrad and a realistic electron doses of 100 eÅ^−2^ or 20 eÅ^−2^.

The 4D-STEM dataset for ePIE reconstruction was generated with the same parameters as for the amorphous case, except for the radiation dose (Table 4).

For iCOM reconstructions, the parameters were set similarly to those for the amorphous case, with the exception that the scan step size was changed so that the spatial frequency corresponding to $2\alpha_{a}$ is properly sampled with 10% headroom. This results in a sampling of $\frac{\lambda }{4\alpha_{a}1.1}$. This was done because above this spatial frequency the CTF remains zero, as shown in Figure 3. The parameters are in Table 5.

**Figure 5:**
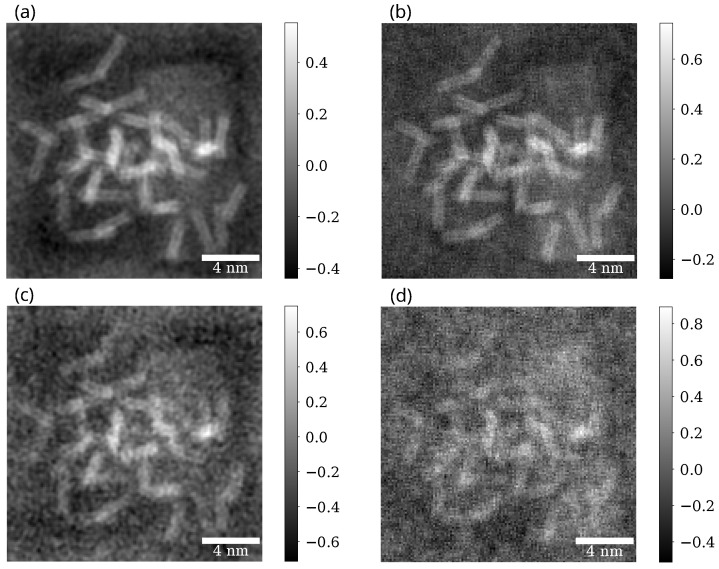
Reconstructions for ePIE (**a**,**c**) and iCOM (**b**,**d**) of datasets with aperture semiangle of 12 mrad and electron doses of 100 eÅ^−2^ (**a**,**b**) and 20 eÅ^−2^ (**c**,**d**). ePIE reconstructions show the phase of the reconstructed object and iCOM reconstructions are multiplied by 2π. All the reconstructions are in the units of radians.

**Figure 6:**
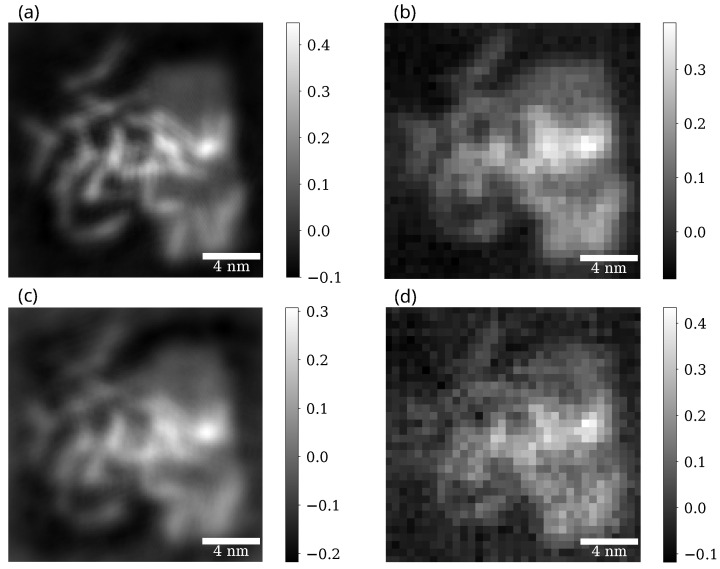
Reconstructions for ePIE (**a**,**c**) and iCOM (**b**,**d**) of datasets with aperture semiangle of 3 mrad and electron doses of 100 eÅ^−2^ (**a**,**b**) and 20 eÅ^−2^ (**c**,**d**). ePIE reconstructions show the phase of the reconstructed object and iCOM reconstructions are multiplied by 2π. All the reconstructions are in the units of radians.

In Figure 5 we can see iCOM and ePIE reconstructions of the sample for aperture semiangle 12mrad and for two electron doses, 100 eÅ^−2^ and 20 eÅ^−2^. Similar reconstructions, but with a different aperture semiangle of 3mrad are shown in Figure 6 again for the two electron doses.

**Figure 7:**
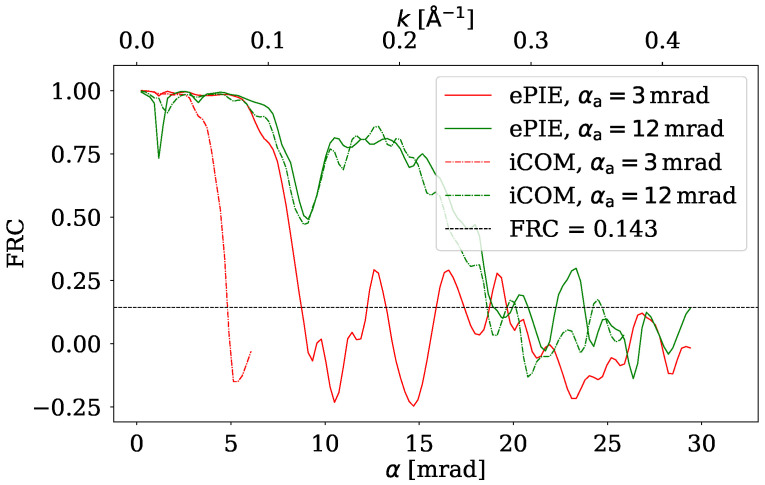
FRC of ePIE and iCOM reconstructions at electron dose of 100 eÅ^−2^ for two aperture angles, with a threshold criterion of FRC = 0.143 shown as a horizontal line.

**Figure 8:**
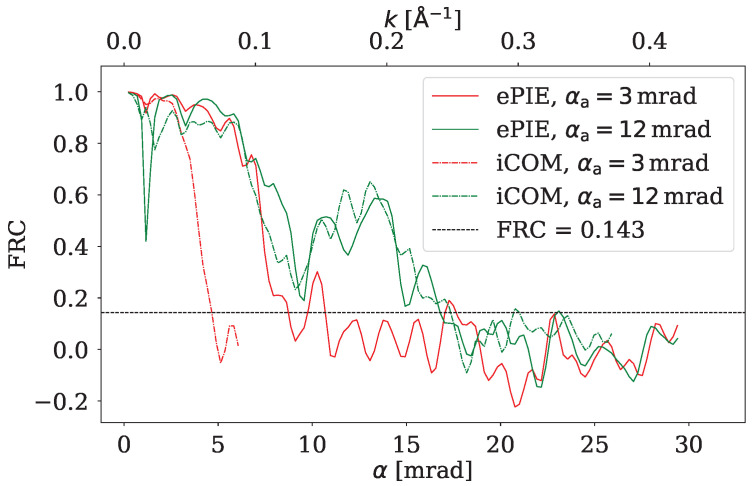
FRC of ePIE and iCOM reconstructions at electron dose of 20 eÅ^−2^ for two aperture angles, with a threshold criterion of FRC = 0.143 shown as a horizontal line.

To investigate the reconstructed images more quantitatively FRC between the reconstructed image and the ground truth was calculated. The results are displayed in Figure 7 and Figure 8 for higher and lower dose, respectively. Solid lines correspond to ePIE, and dash-dotted lines correspond to iCOM. FRC plots also display a FRC=0.143 cutoff criterion, enabling to estimate the resolution of the given reconstruction.

For iCOM the resolution is right below the spatial frequency corresponding to $2\alpha_{a}$, as expected from the CTF of iCOM (Figure 3). This holds true for both radiation doses. For ePIE reconstruction we get a similar FRC curve to that of iCOM when the aperture semiangle is high (12 mrad). However for the lower aperture semiangle (3 mrad) the resolution of ePIE is noticeably above the value limited by $2\alpha_{a}$, revealing details that are not visible in iCOM image at this aperture angle. This difference highlights the ability of ePIE to recover information from diffraction data beyond bright field disc, given the signal to noise ratio is sufficient. This effect is evident in Figure 6a,c where in the bottom right corner of the image are visible nanotubes overlapping graphene sheets. These nanotubes are almost undistinguishable in the iCOM reconstruction, Figure 6b,d.

The reconstructions of iCOM and ePIE at the higher aperture angle, shown in Figure 5, are qualitatively similar for both electron doses. Even the corresponding FRC curves for these two techniques are close to each other. The difference appears to be primarily an amplification of the noise for the iCOM case at high spatial frequencies. This is due to the high Poisson noise and the lack of a bandpass filter in the iCOM reconstruction, which is quite common [25]. For the higher aperture semiangle and for lower electron dose, shown in Figure 6c,d, the graphene sheets are barely visible for both techniques, but nanotubes are still recognizable. iCOM reconstruction is mostly obscured by high-frequency noise.

A note should be made regarding the dwell time and current in the electron beam. If we consider a Timepix3 with the properties discussed in Section 2.1 and the parameters used in the simulations, we can conclude that iCOM would benefit from the event driven detection mode only for the larger aperture of $\alpha_{a}=12\;\mathrm{mrad}$, which accommodates currents of up to 1.6pA. For smaller aperture angle $\alpha_{a}=3\;\mathrm{mrad}$, the current in event driven mode would be only 0.1pA, which would increase the dwell time beyond the frame-based detection of 0.8ms. In fact, it would make sense to reduce the radiation dose below the 20 eÅ^−2^ for the smaller aperture angle, as the iCOM reconstructions would still transfer larger features well enough, and the event driven mode would reduce the dwell time compared to frame-based mode.

For the ePIE datasets the event driven mode would be beneficial only in the case of the larger aperture $\alpha_{a}=12\;\mathrm{mrad}$ and lower dose 20 eÅ^−2^. For the other cases the frame-based detection would be quicker. Since the 4D-STEM dataset for ptychography has a relatively small number of diffraction patterns due to the large scan step size, using frame-based detection with 0.8ms exposure does not pose a huge issue.

## 4. Discussion

We focused on two 4D-STEM phase contrast techniques: iCOM and ptychography (ePIE algorithm). Initially we calculated the CTF for both techniques from reconstructions of an amorphous sample. In the CTFs, we observed that at lower dose, the CTF of ePIE drops to zero around spatial frequency corresponding to $2\alpha_{a}$, which is consistent with [8]. This behavior suggests that the contrast transfer can be adjusted by aperture size to extend to spatial frequencies which are required for observing details of interest. The fact that ePIE can reconstruct CTF ideally under very high doses and that this capability decreases as the dose reduces emphasizes the fact, that the contrast is limited by the electron dose, as noted in [42].

From the analytical CTF of iCOM (8), as well as from CTF calculated on an amorphous sample, we observe that the contrast transfer is also limited to the spatial frequency corresponding to $2\alpha_{a}$. This limitation holds even under very high doses. The strategy of adjusting the aperture angle based on the details of interest is still applicable for iCOM. The benefit of this approach is the same for both ePIE and iCOM: by limiting the aperture, the radiation dose applied on the weakly scattering sample will be invested only in the spatial frequencies of our interest. This allows to balance between resolution and radiation damage, as demonstrated for cryogenic ptychography in [8], where stain-free weakly scattering biological samples has been studied. Authors reconstructed a rotavirus using dose of only 5 eÅ^−2^ with a resolution of 1.5nm according to limit posed by $2\alpha_{a}$.

Beside the reconstruction of the amorphous sample, we also performed reconstructions of a group of nanotubes, Figure 5 and Figure 6. From these, we observe that both iCOM and ePIE yield fairly similar results for each dose: 100 eÅ^−2^ and 20 eÅ^−2^. The main difference is in the noise level, which ePIE naturally suppresses. Furthermore, for the smaller aperture angle $\alpha_{a}=3\;\mathrm{mrad}$ the ePIE algorithm was able to reconstruct spatial frequencies beyond the limit imposed by $2\alpha_{a}$ for weak scattering object. In this case, the dose was still high enough to obtain relatively high scattering outside the bright field disc. This suggests that for the smaller aperture angle, we could reduce the electron dose below the 20 eÅ^−2^ and still obtain a reconstruction with only larger features visible.

## 5. Conclusions

In this study, we explored phase contrast techniques implemented in a SEM in STEM mode using simulations. While phase contrast techniques that do not require a pixelated detector exist, integrating a pixelated detector into a SEM enables the use of 4D-STEM techniques, such as ptychography and iCOM. We observed that these techniques are capable of imaging weakly scattering samples, such as graphene sheets and carbon nanotubes, with good dose efficiency. Moreover, we have demonstrated ptychography’s ability to decouple the probe size from the resolution of the reconstruction, allowing the acquisition of very fine details regardless of geometrical aberrations. Beyond those benefits, repurposing a SEM into a 4D-STEM offers the added advantage of reduced cost compared to a dedicated aberration-corrected STEM, which is significantly more expensive.

However, the results presented here assume a stable microscope and a perfectly coherent beam. In a real-world scenario, various incoherences are inevitable and will degrade the resulting image quality. Minimizing these incoherences is important to achieve the best possible result.

In the future, we will investigate the performance of low-dose phase contrast techniques using data acquired experimentally on a SEM.

## Figures and Tables

**Table 1:** Parameters of the electron beam and detector considered in this study.

$C_{30}$	Energy	Wavelength	Camera Length	Number of Pixels	Pixel Pitch	Maximal Scattering Semiangle
0.88mm	30keV	7pm	240 mm	256 × 256	56μm	30mrad

**Table 2:** Defocus and scan step size for each aperture semiangle $\alpha_{a}$ used in simulations of 4D-STEM dataset for ePIE reconstructions of an amorphous sample.

Dose [eÅ^−2^]	$\alpha_{a}$ [mrad]	Defocus [nm]	Scan Step Size [Å]	Number of Electrons
1300	3	2065	11	$150\times 10^{3}$
1300	6	982	11	$150\times 10^{3}$
1300	12	566	11	$150\times 10^{3}$

**Table 3:** Defocus and scan step size for each aperture semiangle $\alpha_{a}$ used in simulations of 4D-STEM dataset for iCOM reconstructions of an amorphous sample.

Dose [eÅ^−2^]	$\alpha_{a}$ [mrad]	Defocus [nm]	Scan Step Size [Å]	Number of Electrons
*∞*	3	10	1.2	*∞*
*∞*	6	17	1.2	*∞*
*∞*	12	63	1.2	*∞*

**Table 4:** Defocus, scan step size and the number of electrons in each diffraction pattern for each electron dose and aperture semiangle $\alpha_{a}$ used in simulations of 4D-STEM dataset for ePIE reconstructions of nanotubes.

Dose [eÅ^−2^]	$\alpha_{a}$ [mrad]	Defocus [nm]	Scan Step Size [Å]	Number of Electrons
20	3	2065	11	2300
20	12	566	11	2300
100	3	2065	11	11,500
100	12	566	11	11,500

**Table 5:** Defocus, scan step size and the number of electrons in each diffraction pattern for each electron dose and aperture semiangle $\alpha_{a}$ used in simulations of 4D-STEM dataset for iCOM reconstructions of nanotubes.

Dose [eÅ^−2^]	$\alpha_{a}$ [mrad]	Defocus [nm]	Scan Step Size [Å]	Number of Electrons
20	3	10	5.3	570
20	12	63	1.3	35
100	3	10	5.3	2850
100	12	63	1.3	175

## Data Availability

Data are contained within the article.
